# Regulation of water, salinity, and cold stress responses by salicylic acid

**DOI:** 10.3389/fpls.2014.00004

**Published:** 2014-01-23

**Authors:** Kenji Miura, Yasuomi Tada

**Affiliations:** ^1^Faculty of Life and Environmental Sciences, University of TsukubaTsukuba, Japan; ^2^Faculty of Agriculture, Kagawa UniversityKagawa, Japan

**Keywords:** reactive oxygen species, drought tolerance, stomata, salicylic acid, feedback loop

## Abstract

Salicylic acid (SA) is a naturally occurring phenolic compound. SA plays an important role in the regulation of plant growth, development, ripening, and defense responses. The role of SA in the plant–pathogen relationship has been extensively investigated. In addition to defense responses, SA plays an important role in the response to abiotic stresses, including drought, low temperature, and salinity stresses. It has been suggested that SA has great agronomic potential to improve the stress tolerance of agriculturally important crops. However, the utility of SA is dependent on the concentration of the applied SA, the mode of application, and the state of the plants (e.g., developmental stage and acclimation). Generally, low concentrations of applied SA alleviate the sensitivity to abiotic stresses, and high concentrations of applied induce high levels of oxidative stress, leading to a decreased tolerance to abiotic stresses. In this article, the effects of SA on the water stress responses and regulation of stomatal closure are reviewed.

## INTRODUCTION

**FIGURE 1:**
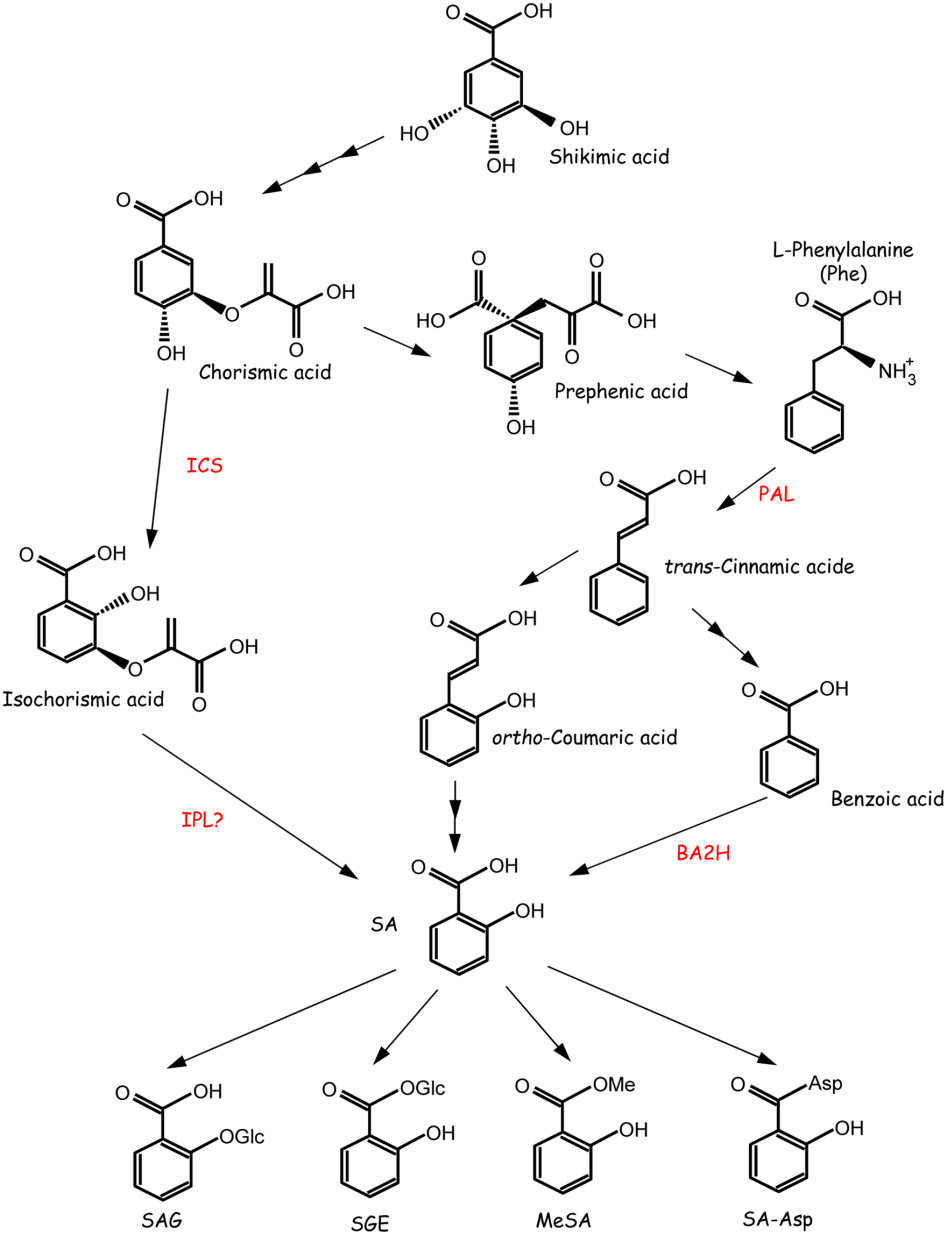
**Proposed pathways for SA biosynthesis and SA modification.** SA is synthesized through the isochorismate (ICS) or phenylalanine ammonia-lyase (PAL) pathways. SA is also converted into several forms. BA2H, benzoic acid-2-hydroxylase; IPL, isochorismate pyruvate-lyase; MeSA, methyl salicylate; SA-Asp, salicyloyl-L-aspartic acid; SAG, salicylic acid 2-*O*-β-glucoside; SGE, salicyloyl glucose ester. The figure is adapted with permission from Dempsey et al. (2011).

Salicylic acid (SA) is involved in the regulation of pathogenesis-related protein expression, leading to plant defense against biotrophic pathogens (Dempsey et al., 2011). It also plays an important role in the regulation of plant growth, development, ripening, flowering, and responses to abiotic stresses (Rivas-San Vicente and Plasencia, 2011; Hara et al., 2012). In general, low concentrations of SA may enhance the antioxidant capacity in plants, but high concentrations of SA may cause cell death or susceptibility to abiotic stresses (Hara et al., 2012). Currently, little information is available about the molecular mechanisms of SA in response to abiotic stresses. The word “salicylic” is derived from *Salix*, which is the Latin name for the willow tree (*Salix alba*). Salicin, the glucoside of salicylic alcohol, was first isolated in 1826 from willow bark, and a large amount of the substance was successfully isolated in 1828. Salicin was then converted into a sugar and an aromatic compound that, upon oxidation, becomes SA. SA, a 2-hydroxybenzoic acid (**Figure 1**), has a colorless crystalline structure and is widely used in organic synthesis, including the synthesis of aspirin, also known as acetylsalicylic acid. Plants generally contain a few micrograms of SA or less per gram of fresh weight (Raskin, 1992), either in a free state or in a glycosylated, methylated, glucose–ester, or amino acid conjugate form (**Figure 1**; Dempsey et al., 2011).

## BIOSYNTHESIS AND SENSORY MECHANISMS OF SA

SA is synthesized via two distinct pathways, the isochorismate (IC) pathway and the phenylalanine ammonia-lyase (PAL) pathway (**Figure 1**). These pathways begin with chorismic acid, which is the end product of the shikimate pathway and is synthesized in the plastid. The major pathway is the IC pathway in *Arabidopsis thaliana, Nicotiana benthamiana*, tomato, and other plants (Wildermuth et al., 2001; Uppalapati et al., 2007; Catinot et al., 2008). Chorismic acid is converted to IC by isochorismate synthase (ICS). *ICS* homologs have been identified in a wide variety of plant species, including tobacco, pepper, tomato, rice, grapevine, soybean, and poplar. *ICS1/SID2* is an important gene in *Arabidopsis* because the mutant accumulates only 5–10% the level of SA compared with wild-type plants (Nawrath and Métraux, 1999; Dewdney et al., 2000). *ICS1/SID2* is up-regulated by not only biotic stresses but also abiotic stresses, including UV light (Kilian et al., 2007), ozone (Ogawa et al., 2005), and drought (Wan et al., 2012). An *Arabidopsis ics1/sid2 ics2* double mutant exhibited an even lower, but not null, level of total SA (Garcion et al., 2008), suggesting the presence of an IC-independent pathway.

Although ICS is conserved in various plant species, the mechanism to convert IC to SA remains unclear. Isochorismate pyruvate lyase (IPL) may catalyze the conversion of IC to SA, given that some bacteria, such as *Pseudomonas aeruginosa* and *Pseudomonas fluorescens*, contain IPL (Serino et al., 1995; Mercado-Blanco et al., 2001). However, no plant gene encoding a protein with IPL activity has been identified. Another pathway is the PAL pathway (**Figure 1**). PAL, the first enzyme in this pathway, deaminates phenylalanine, leading to the production of *trans-*cinnamic acid. *Trans-*cinnamic acid is a precursor for the biosynthesis of diverse phenolic compounds, including lignin, lignans, flavonoids, volatile benzenoid esters, and benzoyl glucosinolates (Weng and Chapple, 2010; Dempsey et al., 2011). Thus, PAL plays an important role as a regulator between primary and secondary metabolism. *Trans-*cinnamic acid is converted to SA via two possible intermediates, *ortho-*coumaric acid and benzoic acid (BA; el-Basyouni et al., 1964; Ellis and Amrhein, 1971; Chadha and Brown, 1974; Yalpani et al., 1993).

SA induces systemic acquired resistance (SAR), which includes global transcriptional reprogramming and immune responses to a broad spectrum of pathogens (Durrant and Dong, 2004). Previous studies identified some SA-binding proteins, such as catalase (Sanchez-Casas and Klessig, 1994), ascorbate peroxidase (APX; Du and Klessig, 1997), methyl SA esterase, and carbonic anhydrase (Slaymaker et al., 2002; Forouhar et al., 2005). These SA-binding proteins have been identified as important SA effector proteins, but genetic evidence suggests that they are not likely to function as bona fide SA receptors (Vlot et al., 2009). According to the large number of studies on SA-insensitive mutants, researchers thought that non-expressor of PR genes 1 (*NPR1*) could be an SA receptor candidate because *npr1* mutant plants exhibit a complete lack of resistance against biotrophic and hemibiotrophic pathogens (Delaney et al., 1995; Cao et al., 1997). Furthermore, the transcriptome analysis of wild-type and *npr1* plants following treatment with BTH, a functional analog of SA, revealed that almost all BTH-responsive genes are under NPR1 control (Wang et al., 2006). NPR1 possesses a bric-a-brac/Pox virus, tramtrack, broad-complex (BTB) domain, an ankyrin repeat and a nuclear localization sequence, but it has no canonical DNA-binding domain (Cao et al., 1997). Although NPR1 has no canonical DNA-binding domain, *NPR1* regulates almost all BTH-responsive genes, suggesting that NPR1 functions as a transcription co-activator in response to SA. However, the NPR1 protein does not show a biologically significant affinity for SA or its derivatives; therefore, another molecule needs to be identified as a receptor for SA.

Recently, the NPR1 paralogs NPR3 and NPR4 were identified as SA receptors that bind specifically to SA with different affinities (Fu et al., 2012). Both of the paralogs interact with the Cullin 3 (CUL3) ubiquitin E3 ligase to recruit NPR1 for proteasome-mediated degradation in a SA concentration-dependent manner. As described above, NPR1 acts as a positive regulator of the SA-mediated defense signaling pathway. When the concentration of SA is low, an NPR4-NPR1 interaction is formed, and NPR4 constitutively promotes the degradation of NPR1 through CUL3-mediated ubiquitylation. Thus, no immune response is activated. An increase in the SA concentration after pathogen attack leads to the binding of SA to NPR4. SA-NPR4 interferes with the NPR4-NPR1 interaction. Because NPR1 is released from NPR4-mediated degradation, free NPR1 can now induce a hypersensitive response (HR), which is a form of programmed cell death that retards pathogenic growth. At very high concentrations, the SA levels are sufficient to bind to NPR3. SA-NPR3 promotes its interaction with NPR1. NPR3 is able to interact with CUL3, leading to ubiquitylation of NPR1. Thus, SA-NPR3-NPR1 formation enhances turnover of NPR1 mediated by proteasome (Fu et al., 2012).

## STOMATAL CLOSURE IS REGULATED BY SA, INDEPENDENT OF THE ABA PATHWAY

The regulation of stomatal guard cells is an adaptive mechanism that helps plants withstand pathogenic infection and extreme environmental conditions, including drought. Stomata play an important role in the uptake of CO_2_ and transpiration. During water deficits, the stomata are closed to slow transpiration and conserve water in the plant, thereby decreasing the CO_2_ supply and leading to a reduction in photosynthesis. Stomatal opening or closure is achieved by the osmotic swelling or shrinking of guard cells, respectively (Liu and Luan, 1998). Plants control the width of the stomatal aperture in response to microorganism invasions (Blatt et al., 1999; Lee et al., 1999; Melotto et al., 2006) and various environmental signals (Hetherington and Woodward, 2003; Cominelli et al., 2005; Liang et al., 2005) as well as phytohormones. Unambiguously, abscisic acid (ABA) plays a substantial role in the regulation of stomatal closure under water stress (Aharoni et al., 1977; Tardieu and Davies, 1992; Schwartz et al., 1995; Leckie et al., 1998). Several studies have suggested that stomatal function is also regulated by auxin (Irving et al., 1992; Lohse and Hedrich, 1992; Gehring et al., 1998), cytokinin (Jewer and Incoll, 1980; Tanaka et al., 2006), ethylene (Desikan et al., 2006; Tanaka et al., 2006), brassinosteroids (Rajasekaran and Blake, 1999; Haubrick et al., 2006), jasmonate (Gehring et al., 1997; Suhita et al., 2004; Munemasa et al., 2007), and SA.

Because stomata are pores in the epidermis, pathogens can enter unchallenged. After an attack by a pathogen, the endogenous SA levels are increased to induce SAR. An increase in endogenous SA levels promotes stomatal closure. This closure is likely caused by the generation of reactive oxygen species (ROS), which is induced by SA (Melotto et al., 2006). The exogenous application of SA also induces ROS, H_2_O_2_, and Ca^2^^+^ accumulation, leading to stomatal closure (Dong et al., 2001; Liu et al., 2003; He et al., 2007). Two major mechanisms have been proposed for the generation of ROS during oxidative burst. One is mediated by plasma membrane NAD(P)H oxidases (Kwak et al., 2003), and another is mediated by cell wall peroxidases (Mori et al., 2001; Khokon et al., 2011). In addition to these enzymes, apoplast amine oxidases (Allan and Fluhr, 1997) and oxalate oxidases are able to generate ROS (Lane et al., 1993). Genetic and pharmacological studies suggest that ABA and methyl jasmonate stimulate NAD(P)H oxidase-mediated ROS production in guard cells (Munemasa et al., 2007; Saito et al., 2008). However, SA induces stomatal closure accompanied by extracellular ROS production that is mediated by salicylhydroxamic acid (SHAM)-sensitive guaiacol peroxidases, intracellular ROS accumulation in guard cells, and K^+^in channel inactivation (Mori et al., 2001; Khokon et al., 2011).

After the contact of pathogenic bacteria with *Arabidopsis* leaves, stomatal closure is induced within 1 h (Melotto et al., 2006). Furthermore, the application of 0.4 mM SA induces rapid stomatal closure within 2 h and a fourfold reduction of stomatal gas exchange in *Arabidopsis* (Melotto et al., 2006). This closure is compromised in the SA-deficient *nahG* and *eds16-2* genotypes, suggesting that SA is required for stomatal defense (Melotto et al., 2006). The SA-accumulating mutants *siz1* (Miura et al., 2005; Lee et al., 2007)*, acd6* (Rate et al., 1999), and *cpr5* (Bowling et al., 1997) exhibit stomatal closure without any treatment (Miura et al., 2013). The stomatal closure of the *siz1* mutant is compromised by the application of SHAM or azide, inhibitors of peroxidases, and not by diphenyliodonium (DPI) chloride, an inhibitor of NAD(P)H oxidase (Miura et al., 2013), suggesting that SA plays a role in the regulation of stomatal closure. Neither the ABA-insensitive mutant *ost1* nor the ABA-deficient mutant *abi3-1* exhibit stomatal closure in response to flg22, a pathogen-associated molecular pattern (PAMP) elicitor or to the bacterial pathogen *Pseudomonas syringae* pv. tomato DC3000, respectively (Melotto et al., 2006). It is possible that positive cross-talk between SA and ABA is required to promote stomatal closure in response to pathogen invasion.

## EFFECTS OF SA ON DROUGHT RESPONSES

Drought is the most common adverse environmental stress that seriously reduces crop productivity. The mechanism for drought avoidance is the maintenance of an adequate supply of water within the plant by growing long roots to reach deep soil moisture (Xiong et al., 2006) or the reduction of transpirational water loss to conserve water (Ackerson and Krieg, 1977). Thus, the stomata play a major role in plant adaptation to drought stress. Drought tolerance refers to the ability of a plant to withstand the loss of water content and regrow when moist conditions return. Resurrection plants have a mechanism to withstand approximately 90% water loss, whereas most other plants can withstand a moderate dehydration of approximately 30% water loss. One characteristic symptom of water deficiency is the mobilization of the starch that is stored in the chloroplasts (Liu et al., 2004). During drought stress, the translocation of carbohydrates decreases, leading to a change in source–sink relationships (Liu et al., 2004). Water deficiency also causes a reduction of nutrient uptake due to the reductions in water migration and the quantity of ions transported by the water and to the retardation of root growth in dry soil (Rahman et al., 1971; Tanguilig et al., 1987). Plants have developed drought avoidance and/or dehydration tolerance to resist drought stresses.

In addition to ABA, SA is involved in the regulation of drought responses. Endogenous SA levels are increased up to fivefold in the evergreen shrub *Phillyrea angustifolia* (Munne-Bosch and Penuelas, 2003). The SA content in barley roots is increased approximately twofold by water deficit (Bandurska and Stroiński, 2005). Furthermore, the SA-inducible genes *PR1* and *PR2* are induced by drought stress (Miura et al., 2013). The induction of SA accumulation may play a role in a protective mechanism during water stress.

However, the effect of SA on drought tolerance remains to be determined because some investigators have reported enhancement of drought tolerance by SA application whereas others have reported a reduction of drought tolerance. Generally, low concentrations of applied SA increase drought tolerance, and high concentrations decrease drought tolerance. As described above, SA induces ROS production in photosynthetic tissues (Borsani et al., 2001). Thus, the application of a high concentration of SA may cause high levels of oxidative stress, leading to decreased abiotic stress tolerance. Both drought tolerance and plant growth are suppressed when a high concentration (2–3 mM) of SA is applied to wheat seedlings, whereas plant growth is enhanced by the application of a low concentration (0.5 mM) of SA (Kang et al., 2012). When wheat seeds were soaked in 100 ppm acetyl SA, the wheat exhibited resistance to drought stress (Hamada, 2001). The application of acetyl SA in the range of 0.1–1 mM also enhanced the drought tolerance of muskmelon seedlings (Korkmaz et al., 2007). The imbibition of tomato and bean seeds in 0.1–0.5 mM SA or acetyl SA increased plant tolerance to heat, chilling, and drought stresses (Senaratna et al., 2000). The treatment of barley with SA decreased the damaging effect of water deficits on the cell membranes in the leaves (Bandurska and Stroiński, 2005). Interestingly, SA treatment increased the ABA content and proline levels in the leaves of barley (Bandurska and Stroiński, 2005). The endogenous SA-accumulating *Arabidopsis* mutants *adr1, myb96-1d, siz1, acd6*, and *cpr5* exhibit both SA-dependent disease resistance and drought tolerance (Bowling et al., 1997; Rate et al., 1999; Grant et al., 2003; Chini et al., 2004; Lee et al., 2007; Seo et al., 2009; Seo and Park, 2010; Miura et al., 2013). The introduction of the pepper pathogen-induced gene *CAPIP2* confers upon *Arabidopsis* resistance to disease and tolerance to drought (Lee et al., 2006). The pretreatment with 0.5 mM SA alleviates substantial water loss from wheat seedlings, leading to an enhancement of drought tolerance (Kang et al., 2012) by influencing the ascorbate–glutathione cycle (Kang et al., 2013).

Proteomics has revealed 37 protein spots that are up-regulated by pretreatment with SA under drought stress. Several stress defense proteins, such as glutathione *S*-transferases, APX, and 2-cysteine peroxiredoxin, are included (Kang et al., 2012), suggesting that SA pretreatment enhances the antioxidant defense system to protect against the oxidative damage caused by drought stress. Proteins involved in ATP synthesis are also up-regulated by SA and drought, most likely due to an increase in growth and to coping with drought stress. In contrast, 21 protein spots, including Rubisco and related enzymes, are down-regulated by SA but up-regulated by treatment with both SA and drought (Kang et al., 2012). Pretreatment with SA enhances photosynthesis under abiotic stress conditions (Singh and Usha, 2003; Syeed et al., 2011). A comparison of microarray data for SA, drought, and H_2_O_2_ treatments and SA-accumulating (*siz1* and *cpr5*) or SA-deficient (*sid2*) mutants revealed that 27 genes in two clusters are up-regulated by SA, drought, and the SA-accumulating mutants *siz1* and *cpr5*. Among these genes, 9 are highly expressed in guard cells (Miura et al., 2013), including *LTI30*. The overexpression of *LTI29* and *LTI30* enhances the accumulation of dehydrins and improves the tolerance to freezing stress (Puhakainen et al., 2004). Because dehydrins play an important role in the tolerance to salt and drought stresses (Brini et al., 2007), *LTI29* and *LTI30* may be involved in the enhancement of drought tolerance.

## SA AND COLD STRESS TOLERANCE

Temperature is also a major factor of abiotic stresses, and it is a key determinant of agricultural yield and crop productivity. The amount and rate of the uptake of water and nutrients are decreased by cold stresses, leading to cell desiccation and starvation. Extreme forms of cold stresses are called freezing stresses and cause ice formation in cell liquids, leading to dehydration and plant death. Cold temperatures promote the accumulation of endogenous free SA and glucosyl SA in *Arabidopsis* shoots, wheat, and grape berry (Scott et al., 2004; Wan et al., 2009; Kosova et al., 2012), suggesting that SA is involved in the regulation of cold responses.

The application of 0.5 mM SA improved the cold tolerance of maize, cucumber, and rice (Kang and Saltveit, 2002). Exogenous SA also decreased freezing injury in the leaves of winter wheat grown under low temperature conditions (Taşgín et al., 2003). Chilling injury in freshly harvested green bell pepper (*Capsicum annuum*) was alleviated by methyl SA and methyl jasmonate (JA) vapors (Fung et al., 2004). This reduction of chilling injury in the green bell pepper was correlated with an increase in the expression of the alternative oxidase (*AOX*) gene induced by methyl SA and methyl JA vapors (Fung et al., 2004). The expression of AOX increased in response to low temperature stresses in rice (Ito et al., 1997), and the capacity of the alternative respiratory pathway and the expression of *AOX* were enhanced under chilling stress (Feng et al., 2008). These observations suggest that an alternative respiratory pathway is involved in the plant response to cold stresses. Lower concentrations of acetyl SA (0.1 mM) or methyl JA (3 μM) significantly improved the seed germination and emergence of sweet pepper (Korkmaz, 2005). Potatoes treated with 0.1 mM SA exhibited freezing tolerance (Mora-Herrera et al., 2005). The application of a 0.5-mM SA solution by spraying the leaves or irrigating the roots of banana seedlings for 1 day improved the chilling tolerance (Kang et al., 2003). When tomato and bean seeds are soaked in aspirin or SA solution (0.1–0.5 mM) before sowing, the cold tolerance of these plants is improved (Senaratna et al., 2000). The hydroponic application of SA or aspirin also increased the chilling tolerance and alleviated the accumulation of both H_2_O_2_ and superoxide radials in the roots and leaves under chilling stress (Wang et al., 2012). SA treatment is effective at alleviating chilling injury, which is one of the most severe postharvest losses of peach fruits. Interestingly, the combination of SA and ultrasound treatment greatly inhibited the chilling injury of peach fruits compared to SA treatment alone (Yang et al., 2012). The application of low concentrations of methyl JA and methyl SA to tomato fruits alleviated the chilling injury and the incidence of decay during low-temperature storage (Ding et al., 2002).

Additionally, high concentrations and the continual application of SA cause severe growth damage and a decrease in the cold tolerance capacity. The *Arabidopsis* SA over-accumulating mutants, such as *acd6, cpr5,* and *siz1* (Bowling et al., 1997; Rate et al., 1999; Lee et al., 2007), are dwarf-like plants due to the reduction of cell elongation and cell proliferation (Rate et al., 1999; Kirik et al., 2001; Miura et al., 2010). Plants from seeds imbibed in a high concentration of SA (1 mM) did not show any alteration of chilling tolerance, whereas low concentrations of SA (0.1–0.5 mM) promoted tolerance to chilling stress in bean and tomato (Senaratna et al., 2000). Winter and spring wheat to which a hydroponic solution of SA was continually applied were severely damaged by freezing temperatures (Horváth et al., 2007), even though the (not continual) application of SA with a foliar spray enhanced the freezing tolerance of winter wheat (Taşgín et al., 2003). The endogenous accumulation of SA by a mutation may cause effects that are similar to those observed after the continual application of SA. The *Arabidopsis* SA-accumulating mutant *cpr1* exhibited a very high accumulation of SA and a strong growth retardation under chilling stress, whereas the growth of *nahG* and *eds5*, in which the accumulation of SA is very low, was greater than that of wild-type plants under low temperature conditions (Scott et al., 2004). The *Arabidopsis cpr1* mutant was damaged by oxidative stress (Scott et al., 2004). It is likely that endogenous SA accumulation triggers production of ROS, which causes cold sensitivity. The other SA-accumulating mutants, *acd6* and *siz1*, were also sensitive to freezing temperatures, whereas the introduction of *nahG* into *acd6* and *siz1* recovered the sensitivity (Miura and Ohta, 2010). The *ice1* mutant, which was originally isolated as a cold-sensitive mutant (Chinnusamy et al., 2003), exhibited an up-regulation of SA-inducible genes (Miura and Ohta, 2010) and enhanced resistance to bacterial pathogens (Zhu et al., 2011). The overexpression of *DEAR1* (DREB and EAR motif protein) enhanced the accumulation of SA and the freezing sensitivity (Tsutsui et al., 2009). *OsWRKY13* enhanced the disease resistance and decreased the salt and cold tolerance in rice (Qiu et al., 2008). These data suggest that temporal application of SA may enhance the cold tolerance but that continual application may decrease this tolerance. Furthermore, *CAMTA3/AtSR1*, which encodes a calmodulin-binding transcription activator, recognizes the *CBF2/DREB1C* promoter to positively regulate the expression of *CBF2/DREB1C* to enhance cold tolerance (Doherty et al., 2009), contributes to the up-regulation of 15% of the cold-inducible genes (Kim et al., 2013). Furthermore, *CAMTA3/AtSR1* binds to the promoter of *EDS1* to repress its expression and disease resistance (Du et al., 2009). These results suggest that cold signaling and SA signaling may be interrelated and that the effect of SA on cold tolerance may be tissue-specific and dependent on the organism, concentration, and period of application.

## SALINITY AND OSMOTIC STRESS TOLERANCES REGULATED BY SA

Salinity stress causes not only cellular sodium toxicity, which destroys the ionic homeostasis and ionic distribution, but also osmotic stress. Salinity stress usually copes with water stress imposed by the low external water potential. More than 20% of irrigated lands are affected by high salt concentrations, and salinity is a common feature of arid and semiarid lands. The endogenous SA level and the activity of the SA biosynthesis enzyme benzoic acid 2-hydroxylase were induced by salinity in rice seedlings (Sawada et al., 2006). The results suggest that SA plays a role in the response to salinity.

The salt-induced decrease in photosynthetic activity and the concentrations of leaf Na^+^, Cl^-^, and H_2_O_2_ were alleviated by the application of SA (0.1 or 0.5 mM) to mung bean (Khan et al., 2010; Nazar et al., 2011). However, high concentrations of SA (1.0 mM) caused growth retardation (Nazar et al., 2011). The exogenous application of SA also improves tolerance to salt stress in several species. The salt tolerance, profitable yield production, and oil content were improved by the application of SA to sunflower plants (Noreen and Ashraf, 2010). Strawberry plants treated with SA exhibited greater growth, as did higher chlorophyll concentrations under salt stress (Karlidag et al., 2009). Tomato plants treated with 0.01 mM SA via root drenching improved the plants’ growth and increased the accumulation of photosynthetic pigments, the K^+^ concentration, and the soluble sugar concentration (Wasti et al., 2012). Pretreatment of tomato with SA in hydroponic culture triggered the accumulation of ABA, leading to an improved acclimation to salt stress (Szepesi et al., 2009). The application of SA improved barley plant growth by promoting protective reactions involving the photosynthetic pigments and maintaining membrane integrity (El-Tayeb, 2005). SA improved wheat plant growth and promoted the accumulation of ABA and proline (Shakirova et al., 2003). The lipid peroxidation and membrane permeability were decreased by SA in maize under salinity stress, leading to the enhancement of plant growth (Gunes et al., 2007). The exogenous application of SA to common bean plants improved plant growth, and the endogenous SA content decreased the growth (Palma et al., 2009). The inhibition of the salt-induced plant growth and photosynthetic capacity of the *Medicago sativa*–*Sinorhizobium meliloti* symbiosis were alleviated by pretreatment with 0.1 and 0.5 mM SA (Palma et al., 2013). The nodule biomass was not affected by salinity in SA-pretreated *Medicago sativa* plants, leading to the maintenance of the nitrogen fixation capacity under salt stress (Palma et al., 2013). The ameliorative effects of SA on salinity stress included a decrease in the Na^+^ content and an increase in the K^+^ concentration in chamomile (Kovacik et al., 2009). The NaCl-induced K^+^ efflux from the roots was prevented by the pretreatment of *Arabidopsis* with 0.01–0.5 mM SA, resulting in enhanced K^+^ retention and improved shoot growth (Jayakannan et al., 2013). High salinity inhibited the germination of *Arabidopsis* seeds. Lower concentrations of SA (<50 μM) reduced the inhibitory effect of high salinity, while higher concentrations of SA (>100 μM) enhanced this effect (Lee et al., 2010). Another report demonstrated that necrotic lesions induced by NaCl treatment were ameliorated in SA-deficient *Arabidopsis nahG* plants (Borsani et al., 2001), in which the glutathione/oxidized glutathione ratio and the ascorbate/dehydroascorbate ratio were greater during salt stress (Cao et al., 2009).

Drought, salinity, and low temperature stress induce osmotic stress, leading to turgor loss. Several reports demonstrate that the application of SA also affects osmotic stress responses. The addition of SA (0.05 mM) to hydroponic solutions containing media and polyethylene glycol (PEG) alleviated the harmful effects of osmotic stress on wheat seedlings (Marcińska et al., 2013). Exogenous SA application positively impacted the colonization of the endophyte *Penicillium resedanum* and relieved the adverse effects of osmotic stress by decreasing losses in *C. annuum* biomass (Khan et al., 2013). The *Arabidopsis wrky54wrky70* mutant, which accumulates high levels of endogenous SA, exhibited tolerance to PEG-induced osmotic stress, which was correlated with improved water retention and enhanced stomatal closure (Li et al., 2013).

## RELATIONSHIP BETWEEN SA AND ROS IN RESPONSE TO ABIOTIC STRESSES, INCLUDING WATER, SALINITY, AND COLD STRESSES

The effects of SA on plant tolerance to abiotic stresses appear to be contradictory. The same SA concentration can enhance the tolerance to one type of stress but decrease the resistance to another type of stress (Németh et al., 2002). Generally, a deficiency or very high level of SA decreases plant tolerance to abiotic stresses. In most low-level SA plants, such as *Arabidopsis* and tobacco, treatment with 0.1–0.5 mM SA is optimal for eliciting the highest level of stress tolerance (Németh et al., 2002; He et al., 2005; Shi et al., 2006). The basal level of total SA containing free SA (active) and SAG (inactive) in *Arabidopsis* or tobacco is 0.25–1 μg/g or less than 0.1 μg/g fresh weight, respectively (Yalpani et al., 1991; Malamy et al., 1992; Nawrath and Métraux, 1999; Wildermuth et al., 2001). The free SA level in *Arabidopsis* is less than 50 ng/g fresh weight (Kiefer and Slusarenko, 2003). Additionally, rice contains higher levels of endogenous SA (5–30 μg/g fresh weight; Yang et al., 2004). Pretreatment with SA at this concentration (0.1–0.5 mM) causes low levels of ROS accumulation (Harfouche et al., 2008). As described above, ROS production mediated by SHAM-sensitive guaiacol peroxidases was induced by SA in guard cells (Mori et al., 2001; Khokon et al., 2011). In addition to peroxidases, other SA effectors are involved in the generation of ROS. APX, catalase, and carbonic anhydrase, which are SA effectors and are involved in scavenging ROS, were inhibited by SA (Chen et al., 1993; Conrath et al., 1995; Durner and Klessig, 1995; Slaymaker et al., 2002). The inhibition of these enzymes by SA induces an increase in the ROS levels. Low ROS levels act as secondary signal molecules to enhance the activities of cellular protective enzymes, including APX, catalase, superoxide dismutase (SOD), guaiacol peroxidase (GPX), glutathione reductase (GR), alternative oxidase (AOX), and heat shock protein (HSP; Janda et al., 1999; Kang and Saltveit, 2002; Taşgín et al., 2003; He et al., 2005; Shi et al., 2006). Non-enzymatic antioxidants such as glutathione, ascorbic acid, carotenoids, and tocopherols can contribute to scavenging ROS (Miyake and Asada, 1994; Telfer et al., 1994, 2003; Shimaoka et al., 2003; Kanwischer et al., 2005; Krieger-Liszkay and Trebst, 2006; Ramel et al., 2012).

**FIGURE 2:**
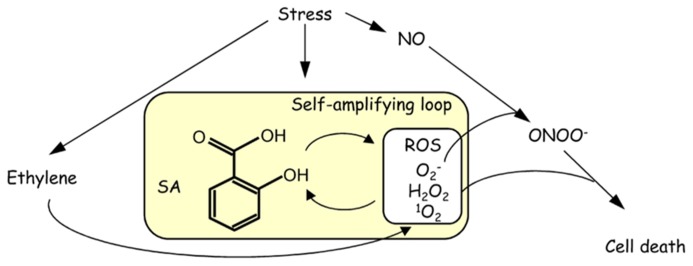
**Schematic model of a self-amplifying feedback loop between SA and ROS in response to stress**.

The application of high concentrations of SA (usually more than 1 mM) induces high levels of ROS accumulation, leading to a decrease in the capacity to scavenge ROS (Mittler, 2002). The over-accumulation of ROS causes oxidative burst, cell death, and a high level of oxidative stress (Leon et al., 1995; Mateo et al., 2006), leading to a decrease in abiotic stress tolerance. Lesion formation due to the accumulation of H_2_O_2_ in the *cat2* mutant, which is impaired in catalase 2 (Queval et al., 2007), is alleviated by the introduction of *sid2*, which is defective in ICS1 (Chaouch et al., 2010). This result indicates that the effect of oxidative stress relies on the IC pathway of SA synthesis. SA and ROS, mainly H_2_O_2_, have been proposed to form a self-amplifying feedback loop in response to abiotic and biotic stresses (Vlot et al., 2009). Stress-induced redox regulation is accompanied by the accumulation of ethylene and nitric oxide (NO), and these molecules participate in the SA-ROS self-amplifying loop (**Figure 2**; Steffens et al., 2013). The reaction of the free radical superoxide with NO results in the generation of the cytotoxic compound peroxynitrite (ONOO^-^), which induces oxidative burst and cell death (Yoshioka et al., 2011). Under abiotic stresses, treatment with an ethylene precursor increases ROS production, and SA-induced cell death is activated by ethylene signaling (Poor et al., 2013). High concentrations of both H_2_O_2_ and SA were involved in the disruption of normal mitochondrial function, leading to decreased electron transport rates and cellular ATP levels (Norman et al., 2004). Intracellular stresses may be recognized by mitochondria, which transduce signals to the nucleus for gene expression alteration.

**FIGURE 3:**
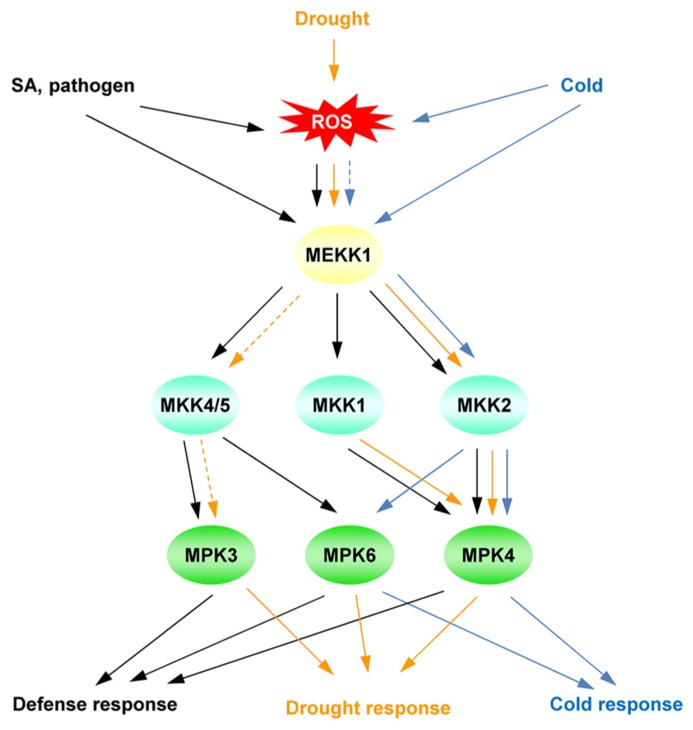
**Simplified model of the MAP kinase cascade, mainly focusing on MPK3/4/6.** The different cascades are distinguished by different colors in the scheme. Solid lines indicate established signaling pathways; dashed lines indicate putative signaling pathways.

A MAP kinase (MPK) cascade may be involved in the transmission of the SA/ROS signal to regulate downstream genes (Rodriguez et al., 2010). Several abiotic stresses primarily activate MPK3, MPK4, and MPK6 in *Arabidopsis* (**Figure 3**; Ichimura et al., 2000; Moon et al., 2003; Ahlfors et al., 2004; Droillard et al., 2004; Teige et al., 2004; Gudesblat et al., 2007). These MPKs are also activated by SA, PAMPs, and ROS (**Figure 3**; Petersen et al., 2000; Asai et al., 2002; Droillard et al., 2004). MAPK cascades are conserved signaling modules in eukaryotes. In a general model, MAP kinase kinase kinases (MEKKs) are activated, and phosphorylate MAP kinase kinases (MKKs), which activate MPKs. MPK6 and MPK3, are the *Arabidopsis* homologs of SA-induced protein kinase (Zhang and Klessig, 1997) and wound-induced protein kinase (Yap et al., 2005), respectively, and are activated by MKK4 and MKK5. The phosphorylation of MPK6 is associated with the application of SA to *Arabidopsis* roots (Mockaitis and Howell, 2000).

Genetically, the MEKK1-MKK1/2-MPK4 cascade plays a negative role in the regulation of defense responses because the loss of function of either MEKK1 or MPK4 prompts the accumulation of SA (Ichimura et al., 2006; Suarez-Rodriguez et al., 2007). Similarly, ROS activate these MAP kinase cascades. ANP1, an *Arabidopsis* NPK1-like protein kinase 1, is activated by H_2_O_2_, leading to the phosphorylation of MPK3/MPK6 in *Arabidopsis* plants (Kovtun et al., 2000). The MEKK1-MPK4 cascade also plays an essential role in ROS metabolism (Nakagami et al., 2006). H_2_O_2_ accumulates in *mekk1* and *mpk4* mutants and activates MEKK1 in protoplasts. Because the MEKK1 protein level is also increased by H_2_O_2_(Nakagami et al., 2006), the MEKK1-MPK4 cascade may be part of a feedback loop that regulates and responds to ROS levels. These MAPK cascades are controlled by both SA and ROS. Because oxidative stress is a common response to biotic and abiotic stresses, ROS homeostasis is a convergence point to evaluate the plant stress status.

## CONCLUDING REMARKS

Salicylic acid plays an important role in the regulation of the abiotic stress responses described above. Application of the appropriate concentration of SA enhances tolerance to abiotic stresses, thereby not only mitigating the damaging effects of abiotic stress tolerance but also enhancing biotic stress tolerance. The important characteristic of SA application is the concentration of applied SA and the method of application, such as foliar spray and hydroponic culture. These methods depend on the plant species; therefore, contradictory results can be reported. Generally, low concentrations or the transient application of SA promotes plant tolerance to abiotic stresses, and high concentrations or the continual application of SA induce inhibitory effects on plant growth and reduce tolerance. It is clear that SA is a very promising compound for the reduction of the abiotic stress sensitivity of numerous plant species.

It remains unclear how SA plays a specific role in abiotic stresses. The accumulation of endogenous SA is induced by several abiotic and biotic stresses. However, how the accumulation of SA is distinguished by each stress is not understood. If the mechanism of how plants distinguish the induction of SA by each stress is understood, this knowledge would contribute to the clarification of the specificity of plant responses to abiotic stresses.

## Conflict of Interest Statement

The authors declare that the research was conducted in the absence of any commercial or financial relationships that could be construed as a potential conflict of interest.
